# SDG-11 and smart cities: Contradictions and overlaps between social and environmental justice research agendas

**DOI:** 10.3389/fsoc.2022.995603

**Published:** 2022-11-29

**Authors:** Ushnish Sengupta, Ulysses Sengupta

**Affiliations:** ^1^Community Economic and Social Development, Algoma University, Brampton, ON, Canada; ^2^Complexity Planning and Urbanism Laboratory, Manchester School of Architecture, Manchester Metropolitan University, Manchester, United Kingdom

**Keywords:** ICT, smart cities, environmental justice, social justice, Sustainable Development Goals (SDGs), SDG-11, sustainable cities, sustainable communities

## Abstract

There is an increased role Information and Communications Technology (ICT) plays in the achievement of Sustainable Development Goals (SDGs). This paper focuses specifically on SDG-11 “Make cities and human settlements inclusive, safe, resilient and sustainable” and how cities are increasingly incorporating ICT toward this goal. The public discourse on Smart Cities suggests economic, social and environmental benefits are possible through the use of Information and Communication Technology (ICT). However, the increased deployment and use of digital infrastructure and processes in the name of sustainability and optimization itself is the focus of a growing body of critical literature on Smart Cities. This mini-review collates critical literature on digital infrastructures and processes related to SDG-11 and Smart Cities to identify areas of significance for further research. Although many Smart City projects discuss sustainability benefits, the distribution of benefits and risks across different communities is rarely examined. An increased use of ICT in Smart City projects can provide environmental benefits to some communities, while shifting the burden of risks to other communities. An increased use of ICT has its own energy and resource impacts that has implications for sustainability beyond the geography of individual cities to global impacts. The lifecycle and supply chain impacts of advanced ICT projects are being identified and documented. The end user of the Smart City projects may benefit significantly from the increased use of ICTs, while the environmental costs are often borne by disparate communities. In some cases, within the same city where a Smart City project is deployed, the inequities in distribution of environmental resources and services are exacerbated by layering new ICT implementations on top of existing socio-economic inequities. Therefore, this paper combines a broad view of Smart City environmental impacts, as well as a deep examination of the intersection of social justice and environmental justice issues to create more wholistic approaches for analysis of governance of Smart City projects. A more wholistic approach for governance of Smart City projects is required that includes combined social justice and environmental justice frameworks, toward achievement of SDG-11 goals.

## Introduction

The 2030 Agenda for Sustainable Development, with it's 17 Sustainable Development Goals (SDGs) is a global call for action toward a more sustainable future. Within the 17 SDGs, SDG 11: “Make cities and human settlements inclusive, safe, resilient and sustainable”, contains several targets (11.1–11.7, 11.a−11.c) and related indicators (including unit of measurement or analysis) for development at an urban or city scale (United Nations Department of Economic and Social Affairs, 2022). Smart City initiatives, with popular discourse suggesting the use of Information and Communication Technology (ICT) and related technological developments can lead to social, economic and environmental benefits (Ismagilova et al., 2019; Raharjana, 2019), initially appear to be in a strong position to engage with sustainable development actions directly related to the SDGs. These ICT driven projects are typically government supported and involve significant investment, infrastructural development and impacts on citizen services. The critical literature on Smart Cities questions the potential benefits in the popular discourse (Kitchin, 2015). There are multiple areas of social and environmental justice that are pointed to as concerns in existing models and manifestations of Smart Cities (Asteria et al., 2020; Loos et al., 2020; Curran and Smart, 2021). Despite the nexus of concerns and actions, smart city research and sustainable development research with a focus on SDG-11 remains largely separate.

Authors researching urbanization (Plieninger et al., 2016), migration economics (Pisarevskaya et al., 2022), culture (Panzera et al., 2021) and technologies (Marvuglia et al., 2020) all converge on cities as the site of contestation for social justice (Harvey, 1997, 2009). The dominant public discourse on SDG 11 typically focuses on physical infrastructure projects in cities, including environmental benefits and risks (Hölscher et al., 2019). Simultaneously, there is a growing literature related to SDG 11 that involves an ICT component, often under the theme of Smart Cities (Bibri and Krogstie, 2017b; Ismagilova et al., 2019). Smart city initiatives in this paper refer to city government-supported projects involving substantial investments in ICT infrastructure and ubiquitous computing projects for real-time monitoring, management and regulation (Kitchin, 2015). This paper draws out specific concepts from this discourse on Smart Cities by analyzing the intersection between social justice and environmental justice. Social justice in this context refers to equitable social and economic outcomes for equity-seeking groups, such as women, racialized communities, older individuals, people with disabilities, and individuals with lower socio-economic status. Environmental justice refers to equitable outcomes for the same equity seeking groups but in terms of sustainability and environmental outcomes (Michalec et al., 2019).

There is a significant body of critical literature on the inequities propagated through urban development, with government led initiatives and infrastructures coming under critical scrutiny (Larsson, 2006; Boyle and Mohamed, 2007; Hopkins, 2010). In parallel, there is an emerging critical literature on Smart Cities, with projects based on promised sustainability and economic benefits being exposed as initiatives with inequitable distribution of benefits and risks (Safransky, 2020; Curran and Smart, 2021; Mouton and Burns, 2021; O'Malley and Smith, 2022). It is the intention of this review to compare the two literatures and conceptually generate the hypothesis that urban infrastructure projects and ICT based Smart City projects have critiques that can be understood through a social justice and environmental justice framework. The findings and arguments in this paper contribute to the intersectional discourse between critical Smart City literature and SDG-11 literature, through identification of critical concepts.

Three key concerns provide a useful framing to the critical literature on ICT related urban development initiatives, namely natural resource usage, distribution of risks and benefits, and energy usage. There are a few authors who discuss this breadth of ICT related environmental and social justice issues. Crawford (2021) discusses the global environmental and social impacts of AI and the geographical distribution of the benefits and risks. Kunkel and Matthess (2020) describes the issues of resource usage, including rare earth minerals and energy, as well as the issue of e-waste. Goel et al. (2021) raise the concerns of both energy use and resource use for sustainable Smart Cities, and point to the growing level of e-waste, recommending a circular economy approach to reducing e-waste and resource usage. The majority of authors focus on a single issue such as increasing energy usage through increasing use of ICT (Wang et al., 2021). First, we identify natural resource usage by ICT projects, where the material supply chain and lifecycle ICT devices and equipment results in significant environmental impacts (Obaid et al., 2021). David and Koch (2019) identify the absence of discourse on critical raw materials in smart city and urban planning literature. Ilankoon et al. (2022) focus attention on the limitations of dependency on the extraction of rare earth minerals. Second, we identify the distribution of environmental benefits and risks with a Smart City project as an area of deficient social and environmental justice. Bauriedl and Strüver (2020) identify the potential risks of ICT projects exacerbating already existing socio-spatial inequalities. Sengupta and Sengupta (2022) summarize case studies on distribution of risks and benefits in Smart Cities including security and discrimination issues. Third, an emerging area of concern is the increased energy usage by large ICT projects, where the consumption of energy by more power intensive processes contributes to global warming (Strubell et al., 2019). Bender et al. (2021) decribe the energy usage by Large Language Models, pointing to new applications of developments in AI resulting in a potential increase in energy use. Obaid et al. (2021) take a more solution oriented approach, in recognizing the increasing energy costs of ICT for smart cities, but advocating for green ICT solutions. As determined by Wang et al. (2021), although the energy usage per device is decreasing over time, the number of ICT devices is increasing at a faster rate than the reduction in energy consumption, resulting in a net increase in overall energy use. In agreement with the broad approach utilized by Crawford (2021) to analyze the social and environmental impacts of AI, an alternative more sustainable governance framework for Smart Cities needs to address the energy usage, material supply chain, and local distribution of benefits and risks for global and local stakeholders. We identify broad environmental impacts of ICT projects where the focus of literature has been too narrow, as well as deep environmental impacts localized within cities where the literature has not been specific on the uneven distribution of risk and benefits.

## Discourse on SDG-11, smart cities, and ICT

### Limited ICT discourse in SDG-11 literature

This section describes the intersectional literature between SDG-11 and ICT. We find a number of limitations in the literature in the discourse of ICT in SDG-11 literature. Thomas et al. (2021) argue that achieving SDG-11 goals requires the additional collection of local and disaggregated data enabled by ICT. In a trend including other scholars arguing for additional collection of data for SDGs, Thomas et al. (2021) do not describe the costs of obtaining additional data, particularly the social costs of datafication (Dencik et al., 2019) and environmental costs (Ensmenger, 2018). Rozhenkova et al. (2019) advocates for SDG-11 related databases, that will enable common data to be compared across cities, therefore recognizing the need for physical infrastructure required for data storage and management. At the same time Rozhenkova et al. (2019) do not address the material aspects of data storage requirements, particularly the social costs (Bouk, 2017) and environmental costs of data storage (Hogan, 2015). The first trend in SDG-11 literature is an absence of consideration of social and environmental impact of ICT based collection and storage of additional data related to SDG-11 indicators, increasing the risks of increased data collection and storage impacts on social and environmental justice.

In the limited SDG-11 literature that does consider environmental impacts of ICT, Allam and Jones (2021) point to the communications aspects of ICT and the physical manifestation of 6G network implementation including devices and connections, and point to ICT as being part of the communication infrastructure required for achievement of SDG-11 for smart and sustainable cities. Allam and Jones (2021) usefully point to the number of devices required and the increased energy demand anticipated by the increased number of devices to achieve the communication infrastructure benefits of 6G networks. The second general trend in SDG-11 literature that does include environmental impacts of ICT is a lack of consideration of the related globalized social justice impacts of ICT (Couldry and Mejias, 2019). There are some notable exceptions: Goel et al. (2021) highlight the bottom-up participatory nature of Smart Cities that utilize resources in a more circular economy, making an explicit connection between social justice and achieving environmental goals. Kaika (2017) provides a strongly critical social justice oriented view of smart cities in the context of the “new urban agenda” connected to SDG-11. Pointing to the environmental risks from natural resource extraction required for ICT devices and systems, Kaika (2017) advocates for a deeper examination of the social and environmental risks of Smart City projects.

Caprotti et al. (2017) take a more critical approach, describing the datafication of citizens as part of the process of collecting data for achieving SDG-11 goals, further detailing the form of datafication as a top-down process where actual citizens views are not known. Caprotti et al. (2017) advocate for more inclusive alternative data measurement approaches involving citizens. Butcher et al. (2021) identifies distribution, participation and recognition aspects of urban governance as being essential in achieving a broad range of SDGs. Masekesa (2021) broadens the concept of participation, identifying the need for public-private partnerships in Zimbabwe to achieve SDG-11 goals. Masekesa (2021) indicates these public-private partnerships include technology infrastructure projects, and that each type of infrastructure has its own partnership requirement. The third trend in SDG-11 literature focusing on social justice aspects of ICT (Schuilenburg, 2015; Pali and Schuilenburg, 2019), is the absence of environmental impacts.

### Environmental discourse in critical smart city literature

The previous section highlighted SDG-11 literature that included discourse on ICT implementation. In this section we broaden the approach of examining literature by highlighting critical Smart City literature with discourse on environmental impacts that include other SDGs in addition to SDG-11. The term “Smart City” is a nebulous term, but in general it has come to represent a city which intensively utilizes Information and Communications Technology (ICT) to achieve its goals (Ingwersen and Serrano-López, 2018). Critical Smart City literature describes smart cities through critiques of both concepts and existent implementations of Smart Cities (Kitchin, 2015). The majority of critical Smart City literature is a sociological critique, pointing to the replication of social inequities by adding a layer of ICT solutions to existing inequitable power structures. In the context of this paper, we utilize the literature to identify both breadth and depth of social and environmental impacts across Smart City related ICT projects.

In a systematic review of Information Systems (IS) literature, Ismagilova et al. (2019) summarize that Smart Cities have the potential to deliver many UN Sustainable Development Goals (SDGs). In the same article, Ismagilova et al. (2019) point to the increasing research on holistic aspects of Smart Cities, “including citizens, quality of living and sustainability” (p. 97). Therefore, although a significant portion of Smart City literature is focused on positive synergies between Smart Cities and SDGs, there is a recognition of a research gap in the combination of social and environmental issues. In a more interdisciplinary literature review, Bibri and Krogstie (2017b) highlight the interdependency between sustainability and Smart Cities, utilizing the term “sustainable smart cities”. Bibri and Krogstie (2017b) usefully point out that Smart Cities may pose risks to environmental sustainability, due to increasing energy usage and lifecycle impact of ICT, similar to previous described articles by Goel et al. (2021) and Obaid et al. (2021). The existing gaps in research identified by Bibri and Krogstie (2017b) include: “There is a need for theory for comparing potential models of smart sustainable city according to their contribution to sustainability goals and smartness targets as an integrated approach.” In a separate article, Bibri and Krogstie (2017a) highlight the importance of power dynamics in the collection and use of data for Smart Cities, again pointing to the negative impacts of energy usage and lifecycle impact of ICT. Therefore, there are some authors engaged in critical Smart City literature usefully identifying both social and environmental impacts of ICT while describing the intersection of Smart Cities and SDGs. The literature on Smart Cities and SDGs are often specific to particular geographical Smart City implementations. In a paper focused on Finnish cities, Ahvenniemi et al. (2017) describe the differences between Smart Cities and sustainable cities, identifying some overlaps as well as differences. In terms of differences, Ahvenniemi et al. (2017) find that Smart Cities in Finland focus on social and economic sustainability, and to a lesser extent environmental sustainability, therefore alluding to a critical gap in the implementation of Smart Cities. In agreement with the systematic review completed by Yigitcanlar et al. (2019), we conclude that not all Smart City projects are environmentally sustainable, but that environmental sustainability is a necessary condition for Smart City projects in achieving broader goals related to SDGs.

There is a related emerging literature that is critical of Smart Cities and their impacts on environmental sustainability. Michalec et al. (2019) describe sustainable Smart City initiatives in the city of Bristol, including electric vehicles and energy retrofits, and conclude that equity issues, although mentioned, were not adequately addressed. Therefore, Smart City projects often focus on achieving environmental goals through the implementation of ICT but fail to consider social equity implications. In an earlier article, Saha and Paterson (2008) emphasize the discourse of sustainable development for cities includes three E's (Environment, Economic development, Equity) and yet the majority of initiatives largely focus on environmental and economic development aspects, and few include concrete equity outcomes. Therefore, there is a pattern of discourse, of discussing social equity goals in Smart City literature, but a lack of mechanisms for measurement of social equity goals at the same level of detail as environmental goals. The issues of environmental justice and social justice need to be addressed as overlapping issues. In other words, increased environmental risks are often experienced by the same groups that face increased social risks of Smart City projects. Levenda et al. (2015) describe the political economy of energy distribution and the “smartening” of the energy grid, concluding that the Smart City projects examined result in additional data collection for marketing with few benefits for citizens. The social inequities generated by additional data collection have been pointed out by Couldry and Mejias (2019) and Dencik et al. (2019). To achieve equity goals in the development of Smart Cities, Sharma et al. (2016) point toward the importance of digital literacy in addressing environmental issues for Smart Cities, and the importance of ensuring equity in digital literacy in reducing the digital divide that exists in many cities. Therefore, some of the Smart City risk mitigation solutions and interventions such as digital literacy can be implemented for equity-seeking groups, and ideally address both social and environmental risks. The digital divide, which is a significant contributor to unequal social justice outcomes, is rarely examined in critical Smart City literature that addresses environmental issues.

## Discussion and conclusion

This paper reviewed the intersectional literature between ICT and SDG-11. The first trend in SDG-11 literature is an absence of consideration for the social and environmental impact of ICT based collection and storage of additional data related to SDG-11 indicators, increasing the risks of uneven social justice. The second general trend in SDG-11 literature that does include environmental impacts of ICT, is a lack of consideration of the globalized social justice impacts of ICT. The third trend in SDG 11 literature focusing on social justice aspects of ICT is the absence of environmental impacts. Next, we examined the environmental discourse in critical Smart City literature which identifies a number of research gaps including social and environmental impacts, and we conclude that not all Smart City projects are environmentally sustainable, but that environmental sustainability is a necessary condition for Smart City projects in achieving broader goals related to SDGs. Usefully identifying gaps requiring additional research, we find examples of discourse on political economy and the digital divide in critical Smart City literature that simultaneously address environmental issues. More broadly, we have identified common areas of concern that positions ICT infrastructures within the sustainable development discourse more immediately, while revealed differences point to areas for further study. This review contributes to SDG research areas on urban inclusion, climate citizenship, social polarization and the equitable distribution of benefits and risks in Smart City projects.

## Future research

One area of future research is the impact of ICT projects and associated rebound effects. Early literature on the net energy usage of ICT, accounting for rebound effects, was optimistic on the net reduction in energy usage (Rodríguez Casal et al., 2005). More recent literature is more pessimistic about the net energy use, including the rebound effect (Marvuglia et al., 2020). In a related development described in this paper, Wang et al. (2021) indicate although the energy usage per device is decreasing over time, the number of ICT devices is increasing at a faster rate than the reduction in energy consumption, resulting in a net increase in overall energy use. Similarly, Allam and Jones (2021) point to the number of devices required and the increased energy demand anticipated by the increased number of devices to achieve the communication infrastructure benefits of 6G networks. Research on rebound effects focused on the social and environmental impacts of ICT is a much needed area of additional knowledge.

A second area of future research is the use of Artificial Intelligence (AI) in the achievement of SDGs. AI is increasingly being used in analysis and modeling for SDGs (Palomares et al., 2021). At the same time, there is a recognition that AI can have both positive and negative impacts on achievement of SDGs (Vinuesa et al., 2020). The areas of concern include increased energy usage by large AI projects (Strubell et al., 2019; Bender et al., 2021), as well as the material impacts of the lifecycle of AI (Crawford, 2021), and political ecnomy of control of AI infrastructure (Whittaker, 2021). Broad research combining the social and environmental impacts of specific ICT projects, such as AI Large Language Models are lacking in literature, whereas these applications are becoming more prevalent portending an increase in energy requirements and social impacts.

A third area of future research is the use of Blockchain technology in the achievement of SDGs. Blockchain technology is increasingly being used in analysis and modeling for SDGs (Parmentola et al., 2022). At the same time, the area of concern is the energy usage by Blockchain technology (Goodkind et al., 2020). The energy usage by popular Blockchains such as Bitcoin rival the energy consumption of entire countries (Beall, 2017). While there are many benefits to utilizing Blockchain technology to develop more sustainable supply chains and energy management systems, the balance between Blockchain simultaneously being part of the solution and part of the problem is a research area of interest.

Fourth, we explicitly identify an underlying tension and tradeoffs between social justice and environmental justice for Smart City projects (Tretter, 2013). Achieving social justice goals does not necessarily accomplish environmental justice goals, and vice versa. Valencia et al. (2019) point out the existence of conflict between SDGs. Future research can explore this argument by identifying conflict within specific SDGs such as SDG-11. Examples of ICT projects which on one hand advance the goals within SDGs and simultaneously effect their own, often unrecognized environmental costs, can be utilized to highlight the conflicts within specific SDG sub-goals.

## Author contributions

Both authors listed have made a substantial, direct, and intellectual contribution to the work and approved it for publication.

## Funding

Funding for the online publication of this article was provided by Algoma University.

## Conflict of interest

The authors declare that the research was conducted in the absence of any commercial or financial relationships that could be construed as a potential conflict of interest.

## Publisher's note

All claims expressed in this article are solely those of the authors and do not necessarily represent those of their affiliated organizations, or those of the publisher, the editors and the reviewers. Any product that may be evaluated in this article, or claim that may be made by its manufacturer, is not guaranteed or endorsed by the publisher.

## References

[B1] Ahvenniemi H. Huovila A. Pinto-Seppä I. Airaksinen M. (2017). What are the differences between sustainable and smart cities? Cities 60, 234–245. 10.1016/j.cities.2016.09.009

[B2] Allam Z. Jones D. S. (2021). Future (post-COVID) digital, smart and sustainable cities in the wake of 6G: digital twins, immersive realities and new urban economies. Land Use Policy 101, 105201. 10.1016/j.landusepol.2020.105201

[B3] Asteria D. Jap J. J. K. Utari D. (2020). A gender-responsive approach: social innovation for the sustainable smart city in Indonesia and beyond. J. Int. Womens Stud. 21, 196–210. Available online at: https://vc.bridgew.edu/jiws/vol21/iss6/12/

[B4] Bauriedl S. Strüver A. (2020). Platform urbanism: technocapitalist production of private and public spaces. Urban Plan. 5, 267–276. 10.17645/up.v5i4.3414

[B5] Beall A. (2017). Bitcoin energy bill matches Ecuador's. New Sci. 236, 8. 10.1016/S0262-4079(17)32143-7

[B6] Bender E. M. Gebru T. McMillan-Major A. Shmitchell S. (2021). On the dangers of stochastic parrots: can language models be too big?, in Proceedings of the 2021 ACM Conference on Fairness, Accountability, and Transparency, 610–623.

[B7] Bibri S. E. Krogstie J. (2017a). On the social shaping dimensions of smart sustainable cities: a study in science, technology, and society. Sustain. Cities Soc. 29, 219–246. 10.1016/j.scs.2016.11.004

[B8] Bibri S. E. Krogstie J. (2017b). Smart sustainable cities of the future: an extensive interdisciplinary literature review. Sustain. Cities Soc. 31, 183–212. 10.1016/j.scs.2017.02.016

[B9] Bouk D. (2017). The history and political economy of personal data over the last two centuries in three acts. Osiris 32, 85–106. 10.1086/693400

[B10] Boyle R. Mohamed R. (2007). State growth management, smart growth and urban containment: a review of the US and a study of the heartland. J. Environ. Plan. Manage. 50, 677. 10.1080/09640560701475337

[B11] Butcher S. Acuto M. Trundle A. (2021). Leaving no urban citizens behind: an urban equality framework for deploying the sustainable development goals. One Earth 4, 1548–1556. 10.1016/j.oneear.2021.10.015

[B12] Caprotti F. Cowley R. Datta A. Broto V. C. Gao E. Georgeson L.. (2017). The New Urban Agenda: key opportunities and challenges for policy and practice. Urban Res. Pract. 10, 367–378. 10.1080/17535069.2016.1275618

[B13] Couldry N. Mejias U. A. (2019). The Costs of Connection: How Data Is Colonizing Human Life And Appropriating It for Capitalism. Stanford, CA: Stanford University Press.

[B14] Crawford K. (2021). Atlas of AI: Power, Politics, and the Planetary Costs of Artificial Intelligence. New Haven, CT: Yale University Press.

[B15] Curran D. Smart A. (2021). Data-driven governance, smart urbanism and risk-class inequalities: security and social credit in China. Urban Stud. 58, 487–506. 10.1177/0042098020927855

[B16] David M. Koch F. (2019). “Smart is not smart enough!” Anticipating critical raw material use in smart city concepts: the example of smart grids. Sustainability 11, 4422. 10.3390/su11164422

[B17] Dencik L. Hintz A. Redden J. Trer,é E. (2019). Exploring data justice: conceptions, applications and directions. Inform. Commun. Soc. 22, 873–881. 10.1080/1369118X.2019.1606268

[B18] Ensmenger N. (2018). The environmental history of computing. Technol. Cult. 59, S7–33. 10.1353/tech.2018.0148 30595595

[B19] Goel R. K. Yadav C. S. Vishnoi S. (2021). Self-sustainable smart cities: socio-spatial society using participative bottom-up and cognitive top-down approach. Cities 118, 103370. 10.1016/j.cities.2021.103370

[B20] Goodkind A. L. Jones B. A. Berrens R. P. (2020). Cryptodamages: monetary value estimates of the air pollution and human health impacts of cryptocurrency mining. Energy Res. Soc. Sci. 59, 101281. 10.1016/j.erss.2019.101281

[B21] Harvey D. (1997). Contested cities: social process and spatial form, in Transforming Cities: Contested Governance and New Spatial Divisions, eds Jewson N. MacGregor S. (London: Routledge), 19–27.

[B22] Harvey D. (2009). Social Justice and the City (Revised edition). Athens: University of Georgia Press.

[B23] Hogan M. (2015). Data flows and water woes: The Utah Data Center. Big Data Soc. 2, 2053951715592429. 10.1177/2053951715592429

[B24] Hölscher K. Frantzeskaki N. McPhearson T. Loorbach D. (2019). Tales of transforming cities: transformative climate governance capacities in New York City, U.S. and Rotterdam, Netherlands. J. Environ. Manage. 231, 843–857. 10.1016/j.jenvman.2018.10.043 30419440

[B25] Hopkins D. (2010). The emancipatory limits of participation in planning: equity and power in deliberative plan-making in Perth, Western Australia. Town Plan. Rev. 81, 55–81. 10.3828/tpr.2009.24

[B26] Ilankoon I. M. S. K. Dushyantha N. P. Mancheri N. Edirisinghe P. M. Neethling S. J. Ratnayake N. P.. (2022). Constraints to rare earth elements supply diversification: evidence from an industry survey. J. Clean. Prod. 331, 129932. 10.1016/j.jclepro.2021.129932

[B27] Ingwersen P. Serrano-López A. E. (2018). Smart City Research 1990-2016. Scientometrics. 117, 1205–1236.

[B28] Ismagilova E. Hughes L. Dwivedi Y. K. Raman K. R. (2019). Smart cities: advances in research—An information systems perspective. Int. J. Inf. Manage. 47, 88–100. 10.1016/j.ijinfomgt.2019.01.004

[B29] Kaika M. (2017). ‘Don't call me resilient again!': The New Urban Agenda as immunology … or … what happens when communities refuse to be vaccinated with ‘smart cities' and indicators. Environ. Urban. 29, 89–102. 10.1177/0956247816684763

[B30] Kitchin R. (2015). Making sense of smart cities: addressing present shortcomings. Cambridge J. Regions Econ. Soc. 8, 131–136. 10.1093/cjres/rsu027

[B31] Kunkel S. Matthess M. (2020). Digital transformation and environmental sustainability in industry: putting expectations in Asian and African policies into perspective. Environ. Sci. Policy 112, 318–329. 10.1016/j.envsci.2020.06.022

[B32] Larsson A. (2006). From equal opportunities to gender awareness in strategic spatial planning: reflections based on Swedish experiences. Town Plan. Rev. 77, 507–530. 10.3828/tpr.77.5.2

[B33] Levenda A. Mahmoudi D. Sussman G. (2015). The neoliberal politics of ‘smart': Electricity consumption, household monitoring, and the enterprise form. Canad. J. Commun. 40, 615–636. Available online at: https://pdxscholar.library.pdx.edu/cgi/viewcontent.cgi?article=1115&context=usp_fac

[B34] Loos E. Sourbati M. Behrendt F. (2020). The role of mobility digital ecosystems for age-friendly urban public transport: a narrative literature review. Int. J. Environ. Res. Public Health 17, 7465. 10.3390/ijerph17207465 33066528 PMC7602187

[B35] Marvuglia A. Havinga L. Heidrich O. Fonseca J. Gaitani N. Reckien D. (2020). Advances and challenges in assessing urban sustainability: an advanced bibliometric review. Renewable Sustain. Energy Rev. 124, 109788. 10.1016/j.rser.2020.109788

[B36] Masekesa L. K. (2021). The potential of public-private partnerships (PPPs) in the pursuit of sustainable development goal 11 in Zimbabwe. Potchefstroom Electron. Law J. 24, 1–43. 10.17159/1727-3781/2021/v24i0a9093

[B37] Michalec A. Hayes E. Longhurst J. (2019). Building smart cities, the just way. a critical review of “smart” and “just” initiatives in Bristol, UK. Sustain. Cities Soc. 47, 101510. 10.1016/j.scs.2019.101510

[B38] Mouton M. Burns R. (2021). (Digital) neo-colonialism in the smart city. Reg. Stud. 55, 1890–1901. 10.1080/00343404.2021.1915974

[B39] Obaid S. Bawany N. Tariq H. binte Tahir, A. Komal N. (2021). Recent trends in green computing, in Innovations in Smart Cities Applications, eds Ben Ahmed M. Rakip Kara? I. Santos D. Sergeyeva O. Boudhir A. A. Vol. 4 (Springer International Publishing), 1071–1083. Available online at: https://www.springerprofessional.de/en/recent-trends-in-green-computing/18871760

[B40] O'Malley P. Smith G. J. (2022). ‘Smart' crime prevention? Digitization and racialized crime control in a Smart City. Theoretical Criminol. 26, 40–56. 10.1177/1362480620972703

[B41] Pali B. Schuilenburg M. (2019). Fear and fantasy in the smart city. Crit. Criminol. 28, 775–788. 10.1007/s10612-019-09447-7

[B42] Palomares I. Martínez-Cámara E. Montes R. García-Moral P. Chiachio M. Chiachio J.. (2021). A panoramic view and swot analysis of artificial intelligence for achieving the sustainable development goals by 2030: progress and prospects. Appl. Intelligence 51, 6497–6527. 10.1007/s10489-021-02264-y 34764606 PMC8192224

[B43] Panzera E. de Graaff T. de Groot H. L. F. (2021). European cultural heritage and tourism flows: the magnetic role of superstar World Heritage Sites. Paper Reg. Sci. 100, 101–122. 10.1111/pirs.12562

[B44] Parmentola A. Petrillo A. Tutore I. De Felice F. (2022). Is blockchain able to enhance environmental sustainability? A systematic review and research agenda from the perspective of Sustainable Development Goals (SDGs). Bus. Strategy Environ. 31, 194–217. 10.1002/bse.2882

[B45] Pisarevskaya A. Scholten P. Kaşl,i Z. (2022). Classifying the diversity of urban diversities: an inductive analysis of European cities. J. Int. Migrat. Integrat. 23, 655–677. 10.1007/s12134-021-00851-z

[B46] Plieninger T. Draux H. Fagerholm N. Bieling C. Bürgi M. Kizos T.. (2016). The driving forces of landscape change in Europe: a systematic review of the evidence. Land Use Policy 57, 204–214. 10.1016/j.landusepol.2016.04.040

[B47] Raharjana I. K. (2019). A systematic literature review of environmental concerns in smart-cities. IOP Conf. Series Earth Environ. Sci. 245, 012031. 10.1088/1755-1315/245/1/012031

[B48] Rodríguez Casal C. Van Wunnik C. Sancho L. D. Burgelman J. C. Desruelle P. (2005). How will ICTs affect our environment in 2020? Foresight J. Futures Stud. Strategic Think. Policy 7, 77–87. 10.1108/14636680510581330

[B49] Rozhenkova V. Allmang S. Ly S. Franken D. Heymann J. (2019). The role of comparative city policy data in assessing progress toward the urban SDG targets. Cities 95, 102357. 10.1016/j.cities.2019.05.026

[B50] Safransky S. (2020). Geographies of algorithmic violence: redlining the smart city. Int. J. Urban Reg. Res. 44, 200–218. 10.1111/1468-2427.12833

[B51] Saha D. Paterson R. G. (2008). Local government efforts to promote the ‘three es' of sustainable development: Survey in medium to large cities in the united states. J. Plan. Educ. Res. 28, 21–37. 10.1177/0739456X08321803

[B52] Schuilenburg M. (2015). Behave or be banned? Banning orders and selective exclusion from public space. Crime Law Soc. Change 64, 277–289. 10.1007/s10611-015-9593-3

[B53] Sengupta U. Sengupta U. (2022). Why government supported smart city initiatives fail: examining community risk and benefit agreements as a missing link to accountability for equity-seeking groups. Front. Sustain. Cities 4, 960400. 10.3389/frsc.2022.960400

[B54] Sharma R. Fantim A. -R. Prabhu N. Guan C. Dattakumar A. (2016). Digital literacy and knowledge societies: A grounded theory investigation of sustainable development. Telecommun. Policy. 40, 628–643. 10.1016/j.telpol.2016.05.003

[B55] Strubell E. Ganesh A. McCallum A. (2019). Energy and policy considerations for deep learning in NLP. *arXiv*. Available online at: https://arxiv.org/abs/1906.02243

[B56] Thomas R. Hsu A. Weinfurter A. (2021). Sustainable and inclusive – Evaluating urban sustainability indicators' suitability for measuring progress towards SDG-11. Environ. Plan. B Urban Anal. City Sci. 48, 2346–2362. 10.1177/2399808320975404

[B57] Tretter E. M. (2013). Contesting Sustainability: ‘SMART Growth' and the Redevelopment of Austin's Eastside. Int. J. Urban. Region. Res. 37, 297–310.

[B58] United Nations Department of Economic Social Affairs, (2022). Goal 11 Make Cities and Human Settlements Inclusive, Safe, Resilient and Sustainable. Available online at: https://sdgs.un.org/goals/goal11 (accessed September 22, 2022).

[B59] Valencia S. C. Simon D. Croese S. Nordqvist J. Oloko M. Sharma T.. (2019). Adapting the sustainable development goals and the new urban agenda to the city level: initial reflections from a comparative research project. Int. J. Urban Sustain. Dev. 11, 4–23. 10.1080/19463138.2019.1573172

[B60] Vinuesa R. Azizpour H. Leite I. Balaam M. Dignum V. Domisch S.. (2020). The role of artificial intelligence in achieving the sustainable development goals. Nat. Commun. 11, 233. 10.1038/s41467-019-14108-y 31932590 PMC6957485

[B61] Wang L. Chen Y. Ramsey T. S. Hewings G. J. D. (2021). Will researching digital technology really empower green development? Technol. Soc. 66, 101638. 10.1016/j.techsoc.2021.101638

[B62] Whittaker M. (2021). The steep cost of capture. Interactions 28, 50–55. 10.1145/3488666

[B63] Yigitcanlar T. Kamruzzaman M. Foth M. Sabatini-Marques J. da Costa E. Ioppolo G. (2019). Can cities become smart without being sustainable? A systematic review of the literature. Sustain. Cities Soc. 45, 348–365. 10.1016/j.scs.2018.11.033

